# “Sisters Are Doin’ It for Themselves”: Gender Differences in Singles’ Well-Being

**DOI:** 10.1177/19485506241287960

**Published:** 2024-10-24

**Authors:** Elaine Hoan, Geoff MacDonald

**Affiliations:** 1 University of Toronto, Ontario, Canada

**Keywords:** singlehood, well-being, gender, MNLFA

## Abstract

The experience of singles has been largely overlooked in relationship science, garnering a need for understanding correlates of singles’ well-being. Gender is an important focus of well-being research, and qualitative work on singlehood has suggested that men and women may have experiences of singlehood that differ in important ways. In this study (*N* = 5941; 50% men; *M*_age_ = 31.74), we provide the first comprehensive, descriptive profile of gender differences on a suite of variables with important ties to well-being in singlehood; satisfaction with relationship status, life satisfaction, sexual satisfaction, and desire for a partner. Our results suggest that single women, on average, report higher levels of satisfaction with relationship status, life satisfaction, sexual satisfaction, and lower desire for a partner. Exploratory analyses showed significant gender interactions with age and ethnicity. Overall, these findings suggest that women are, on average, happier in singlehood than men.

Relationship science has predominantly occupied itself with examining the experiences of those whose relationship status is partnered. However, singlehood is also a relationship status yet has only recently begun to receive systematic research attention. By overlooking the experiences of singles, researchers fail to account for an important population crucial to our understanding of relationship science as a whole. Given an increasing recognition of the variety of experiences of singlehood (Girme et al., 2023), increasing understanding of when and for whom singlehood optimizes well-being is an important research priority.

Demographic characteristics are one key variable in understanding variability in well-being. One demographic characteristic that has been of long-standing interest in psychological research is gender; a large body of work has documented a persistent gender gap in subjective well-being, often demonstrating that women report greater well-being than men (Guven et al., 2012; Zweig, 2015). Gender differences have also been a pervasive feature of romantic relationship research (Karantzas et al., 2011), with work showing that women report lower sexual satisfaction (Carpenter et al., 2009) and lower relationship satisfaction over time (Kurdek, 2005). Given that gender plays a role in well-being in romantic relationships, it seems reasonable to ask whether gender is also an important variable to consider for singles. Indeed, one common stereotype is that single women are unattractive and miserable “spinsters” (Ward, 2020) while single men are labeled as romantically desired “bachelors” (Nanik et al., 2018).

Examining gender is especially pertinent for singles who are a heterogeneous group with differences tied to various demographics. For example, Kislev and Marsh’s (2023) review shows that singles exhibit diversity across various domains like age, ethnicity, and sexuality, which could result in diverse experiences of singlehood tied to well-being. Insights from qualitative work have also separately highlighted unique themes among single men and women’s experiences. Whereas men describe experiences with navigating traditional masculinity (Mrozowicz-Wrońska et al., 2023), women describe dealing with expectations of marriage and motherhood (Simpson, 2016). Thus, investigating whether gender could play a role in singles’ well-being seems important for providing insight into the experiences of singles.

An important issue in considering the role of gender differences in singles’ well-being is which well-being indicators are most important. One of the most widely examined well-being indicators is life satisfaction, defined as the subjective assessment of one’s overall quality of life (Shin & Johnson, 1978), which is often used synonymously with well-being (Diener et al., 1999). Some existing work has examined life satisfaction differences across gender for singles. For example, both Stronge et al. (2019) and Hill Roy et al. (2023) found that single women reported greater life satisfaction than single men. On the contrary, Oh et al. (2022) examined 3,439 singles’ well-being across a 10-year period and showed no significant associations between gender and life satisfaction. Similarly, Adamczyk (2017) showed no significant gender differences in 556 Polish singles’ life satisfaction, while Yan and colleagues’ (2024) also showed no gender differences in life satisfaction in 199 Malaysian singles. Thus, there is mixed evidence suggesting that if there is a difference at all, it is in the direction of single women having higher life satisfaction.

In addition to life satisfaction, singlehood research has also examined relationship status satisfaction as another important well-being indicator. Relationship status satisfaction can be defined as the level of satisfaction with one’s current relationship status as a single or partnered individual (Lehmann et al., 2015) and appears more sensitive to whether someone is single or partnered than life satisfaction (Hoan & MacDonald, 2024). Research examining gender differences in status satisfaction is scarce with mixed conclusions. On the contrary, Adamczyk’s (2017) study showed no gender differences in satisfaction with relationship status. However, two studies measuring the highly related construct of satisfaction with singlehood demonstrated in Polish and German samples that women reported greater satisfaction with singlehood than men (Ochnik & Slonim, 2020; *N* = 512; Ochnik, 2023; *N* = 278). Overall, while relationship status satisfaction is an important well-being indicator, especially in the singlehood context, gender differences on this variable remain unclear.

Moreover, sexual satisfaction, or the fulfillment of sexual needs, has been linked to higher well-being in singlehood, but also higher odds of entering a committed relationship (Park et al., 2021). In romantic relationships, research has suggested that men report greater sexual satisfaction than women (Mark et al., 2015; McNulty et al., 2016). Park and MacDonald (2022) found in one relatively young sample that both single and partnered women were more sexually satisfied than men, but these effects did not replicate in an older sample. Furthermore, in one national Norwegian sample, Fischer (2023; *N* = 1,075) found comparable levels of sexual satisfaction across single men and women. Thus, while sexual satisfaction is an important well-being indicator and predicts singles’ well-being, gender differences in sexual satisfaction are unclear.

Arguably one of the greatest predictors of well-being is motivation (Diener, 1984). Motivations to be single or partnered have been a central theme in the singlehood and well-being literature (MacDonald & Park, 2022; Stein, 1975). One relevant motivational construct is the desire for a partner, or the extent to which one wants to be romantically partnered. Existing research has drawn a clear negative relationship between high partner desire and singles’ well-being (Kislev, 2022; MacDonald & Park, 2022; *N* = 1,930). While a plethora of research has examined gendered preferences in the types of desired romantic partners (Eastwick & Finkel, 2008), less is known about gender differences in who wants to be partnered at all. One study examining older Black Americans showed that Black women were less likely to desire a romantic relationship (Mouzon et al., 2020; *N* = 766). Along similar lines, Sprecher and Felmlee (2021; *N* = 616) found that single men reported a greater fear of being single, suggesting that they may have a greater desire to be partnered than single. Similarly, one study found that single men reported the greatest desires for romance during the COVID-19 pandemic (Tessler et al., 2024). Altogether, the desire for a partner is a key predictor of singles’ well-being, yet existing research does not allow for conclusions about overall gender differences in who wants to be partnered more.

Overall, existing research suggests that there may be gender differences in singles’ well-being and desire for a partner, but some of this work shows mixed results or shows a lack of gender differences in singles’ well-being altogether. Thus, it remains unclear, descriptively, whether single men and women differ in their well-being and desire for partnership. Thus, our priority in this paper is descriptive information, but there are important theoretical explanations worth considering for gender differences in singles’ well-being which we will explore in the Discussion.

Importantly, there are several limitations of the existing research that interfere with our ability to draw conclusions and begin to build theory about gender differences in singles’ well-being. First, much of this work employs a piecemeal approach of examining well-being indicators separately. However, to capture a more holistic view of singles’ overall well-being, a more comprehensive suite of well-being measures should be employed. Second, some existing work suffers from small sample sizes which could result in a lack of statistical power, and subsequently, unreliable conclusions. Third, while existing qualitative work provides important insights into men and women’s unique experiences of singlehood, it remains unclear how experiences of singlehood might directly compare across genders. Finally, one important limitation of existing work is the focus on between-group comparisons across single and partnered individuals. Given that singles are a highly heterogenous demographic, it is important to also conduct within-group assessments of singles’ experiences (Park et al., 2024). Thus, this study aimed to examine gender differences in singles’ well-being, including life satisfaction, relationship status satisfaction, and sexual satisfaction, and the desire for a partner. To do so, we combined and analyzed 10 existing data sets that were previously collected for other research aims (see Supplementary Table S1 for the study descriptions of each data set).

## Methods

### Transparency and Openness

Data collection was approved by the Research Ethics Board at the University of Toronto. We aggregated data collected for purposes other than the present analyses. The samples analyzed in this study were used in previous published studies (Hill Roy et al., 2023; Hoan & MacDonald, 2024; MacDonald & Park, 2022; Park et al., 2022, 2023a, 2023b; Park & MacDonald, 2022, 2023); however, the primary analyses conducted in this study are novel. The analyses conducted in this study were not preregistered as data collection had already occurred at the study’s conception and the authors were already familiar with the data sets. Thus, all results are exploratory and all interpretations of the data are post hoc. Study materials, including data and code for the primary analyses, can be found at https://osf.io/qydmb/.

### Participants

The present analyses were based on 10 different samples of singles recruited for different study purposes. Participants were recruited through the online crowdsourcing platform, Prolific Academic, between April 2020 and April 2023. Participants were required to be at least 18 years of age (*n* = 2 excluded) and report not being in a romantic relationship at the time of the study (*n* = 74 excluded) to be eligible to participate. Our final sample consisted of 5,941 participants (2,890 men, 2,831 women, and 29 individuals identifying as nonbinary) who were 31.74 years old on average (*SD* = 11.64, range = 18–75; see Table 1 for participant characteristics by sample and Supplemental Table S1 for full demographics by sample). Based on a power analysis conducted in G*power, this sample provided >95% power to detect a small effect size (*f*^2^ = 0.02).

**Table 1:** Participant Characteristics per Sample

Sample	Data collection	Gender	Age (*SD*)	Sexual orientation	Ethnicity	Study description
Sample 1 (*n* = 534)	April 2023	291 M, 231 W, 9 N	29.26 (9.147)	77% Heterosexual	–	Study about sexual connections in singlehood
Sample 2 (*n* = 380)	March 2023	140 M, 209 W, 9 N	29.31 (9.96)	71% Heterosexual	–	Study about personality and well-being
Sample 3 (*n* = 475)	January 2023	252 M, 217 W, 6 N	28.73 (8.93)	78% Heterosexual	67% White	Study about personality and well-being
Sample 4 (*n* = 747)	January 2021	376 M, 371 W	38.82 (11.66)	86% Heterosexual	–	Study about singles’ time use and well-being
Sample 5 (*n* = 200)	November 2018	114 M, 84 W, 1 N	33.73 (10.61)	87% Heterosexual	73% White	Study about beliefs about relationships and singlehood
Sample 6 (*n* = 489)	June 2021	243 M, 245 W	23.40 (6.24)	77% Heterosexual	58% White	Study about motivations for dating
Sample 7 (*n* = 811)	November 2021	416 M, 390 W	24.64 (6.49)	77% Heterosexual	31% White	Study about motivations for dating
Sample 8 (*n* = 525)	January 2021	261 M, 264 W	24.67 (8.59)	78% Heterosexual	71% White	Study about singles’ ideal partner characteristics
Sample 9 (*n* = 864)	April 2020	428 M, 434 W, 2 N	38.49 (11.76)	81% Heterosexual	74% White	Study about profiles of singles
Sample 10 (*n* = 937)	December 2020	462 M, 473 W, 2 N	37.76 (11.66)	81% Heterosexual	78% White	Study about singles’ social goals

*Note*. Please see Supplementary Table S1 for full participant characteristics by sample. M = man, W = woman, *N* = nonbinary.

### Measures

Participants were asked to complete a variety of questionnaires that included but were not limited to the measures listed below (see Table 2 for the descriptives of each measure, Supplementary Table S2 for the descriptives and reliabilities of each scale for each sample, and the Supplementary Figure S1 for a visualization of means across the study time periods). Measures were selected for analysis if they were present across enough studies to provide reliable results.

**Table 2:** Descriptive Statistics of Key Measures

Key Measures	Mean	*SD*	Range
Relationship status satisfaction	1.52	0.83	0–3
Life satisfaction	3.55	1.45	1–7
Sexual satisfaction	2.65	1.68	1–7
Desire for a partner	4.22	1.62	1–7

*Note*. *SD* = standard deviation.

#### Satisfaction With Relationship Status

The Satisfaction with Relationship Status Scale (Lehmann et al., 2015) assesses satisfaction with current relationship status and was designed to provide equivalence of measurement across singles and partnered people. The scale is comprised of four items (e.g., How happy are you with your current status?”; *α*s > 0.88) rated on a 4-point scale ranging from 1 (not at all) to 4 (to a great extent). Participants specifically asked to think about their own relationship status was answering the questions.

#### Life Satisfaction

Life satisfaction was assessed using the Satisfaction with Life Scale (Diener et al., 1985). This scale consists of five items (e.g., In most ways my life is close to my ideal; *α*s > 0.88) rated on a 7-point scale ranging from 1 (*strongly disagree*) to 7 (*strongly agree*).

#### Sexual Satisfaction

Sexual satisfaction was assessed using the Satisfaction with Sex Life Scale—Revised (Park & MacDonald, 2022). This scale is comprised of four items (e.g., “In most ways, my sexual life is close to my ideal”; *α*s > 0.95). All items were rated on a 7-point scale ranging from 1 (not at all) to 7 (extremely).

#### Desire for a Partner

Desire for a partner was assessed using the Desire for a Partner Scale. This is a face-valid measure designed by our lab to assess individual differences in how much an individual wants a romantic partner. The validity of this scale has been suggested by moderate to high associations with relevant variables such as the fear of being single (Spielmann et al., 2013) and commitment readiness (Hadden et al., 2018). This scale is comprised of five items, “I want to have a romantic partner,”“My preference for my life right now is to be in a romantic relationship,”“I don’t want to be in a romantic relationship right now” (reverse coded), “My ideal right now is to be in a romantic relationship,” and “Being in a romantic relationship is not something I really value at the moment” (reverse coded); αs > 0.91.

### Analytic Plan

We employed the integrative data analysis (IDA) approach (Curran & Hussong, 2009) in which we pooled raw data from 10 different samples of singles to create a unified data set. Prior to conducting the IDA, we employed a moderated nonlinear factor analysis (MNLFA; Curran et al., 2014) to create commensurate scale scores for relationship status satisfaction, life satisfaction, sexual satisfaction, and desire for a partner. The MNLFA allows us to generate factor score estimates that account for differences in factor mean, variances, and differential item functioning (DIF) in factor loadings and intercepts across covariates. These factor score estimates were used for all analyses described below. Although we logically harmonized similar scales across studies, there remains the potential for heterogeneity across samples, such as in measurement properties. For example, as shown in Table 1 (see full sample demographics in Supplementary Table S1), Sample 9 was collected shortly after COVID-19 was declared a pandemic, which could have affected well-being levels. As such, there may exist heterogeneity across data collection periods (Curran & Hussong, 2009) that could introduce systematic bias. Thus, we used sample membership as a covariate. Our final MNLFA model demonstrated several differences across samples. For instance, sample 5 demonstrated lower mean impact scores for life satisfaction and relationship status satisfaction, while sample 2 demonstrated sexual satisfaction scores. We followed Gottfredson and colleagues’ (2019) steps to run the aMNLFA. We first used the aMNLFA package (Cole et al., 2021; Gottfredson et al., 2019) to generate syntax for the models which were subsequently run on Mplus version 8 (Muthén & Muthén, 2017). The remaining analyses were conducted using R, version 4.3.3 (R Core Team, 2021; see Supplementary Materials for R packages used).

We first fitted separate linear regression models with gender as the predictor and relationship status satisfaction, life satisfaction, sexual satisfaction, and desire for a partner as the outcome variables. Continuous variables were centered for all analyses. Only a binary gender distinction was used (such that men = 1 and woman = 2) because samples sizes for individuals identifying as nonbinary or other (*n* = 29) were statistically underpowered (see Table 1 for gender counts per sample). Notably, samples 7 and 8 assessed sex (i.e., male or female), rather than gender. Although we note that sex and gender are distinct, we will be using sex for those samples as our closest assessment of gender. Second, we tested for gender moderations with age for all four outcome variables to examine intersectional effects and further fit regression models with age and ethnicity as predictors for each outcome. We excluded mixed-ethnicity participants from these analyses (*n* = 59 excluded) due to a lack of power.

## Results

### Gender and Well-Being Associations

As described in the analytic plan, we conducted separate linear regressions with gender as a predictor of the well-being indicators (see Table 3 for correlations between all variables). The strongest effect emerged when relationship status satisfaction was the outcome such that single women reported significantly higher relationship status satisfaction (*b* = 0.30, *t* = 11.59, *p* < .001, 95% CI = [0.26, 0.32], *R*^2^ = 0.02). Our findings also revealed that single women reported significantly higher life satisfaction (*b* = 0.10, *t* = 3.88, *p* < .001, 95% CI = [0.08, 0.14], *R*^2^ = 0.003) and sexual satisfaction (*b* = 0.08, *t* = 2.90, *p* = .004, 95% CI = [0.06, 0.13], *R*^2^ = 0.002). For gender associations with desire for a partner, single women reported significantly lower desire for partnership (*b* = –0.22, *t* = –7.72, *p* < .001, 95% CI = [–0.24, –0.18], *R*^2^ = 0.01).

**Table 3:** Zero-Order Correlations Between Variables

Variable	1	2	3	4	5	6
1. Gender	–					
2. Age	0.04**	–				
3. Relationship status satisfaction	0.14***	0.04**	–			
4. Life satisfaction	0.06***	–0.05***	0.50***	–		
5. Sexual satisfaction	0.06**	–0.05**	0.52***	0.39***	–	
6. Desire for a partner	–0.11***	–0.16***	–0.53***	–0.10***	–0.41***	–

Note:***p < .01, **p < .001.

To directly examine differences across genders, we further conducted independent samples *t*-test analyses which similarly showed that single women demonstrated significantly greater well-being across all four indicators including relationship status satisfaction, *t*(5,862) = –11.58, *p* < .001, *d* = –0.30, life satisfaction, *t*(5,893) = –4.23, *p* < .001, *d* = –0.11, sexual satisfaction, *t*(3,003) = –2.41, *p* = .02, *d* = –0.09, and a desire for a partner, *t*(4,803) =7.60, *p* < .001, *d* = 0.21.

### Intersectional Effects With Gender

#### Gender and Age

To examine intersectional effects between gender and age as well as gender and ethnicity, we conducted moderation analyses examining the interactions between gender alongside age or ethnicity with the well-being outcome of relationship status satisfaction, life satisfaction, sexual satisfaction, and desire for a partner, as shown in Table 4 (see Supplementary Table S3 for full results). When examining the interaction between gender and age, significant two-way interactions emerged for life satisfaction and sexual satisfaction. Specifically, simple slopes analyses revealed that older single men reported lower life satisfaction (*b* = –0.01, *t* = 4.77, *p* < .01, *R*^2^ = 0.01) as well as lower sexual satisfaction (*b* = –0.01, *t* = –2.51, *p* < .05, *R*^2^ = 0.01) while age was not significantly related to life satisfaction (*b* = 0.00, *t* = –0.36, *p* = .72) or sexual satisfaction (*b* = 0.00, *t* = 0.98, *p* = .33) for older single women. No significant interaction emerged between gender and age for partner desire.

**Table 4:** Gender by Age and Ethnicity Interactions

	Satisfaction with relationship status				Life satisfaction				Sexual satisfaction				Desire for a partner			
Predictor	*b*	β	*t*	*p*	*b*	β	*t*	*p*	*b*	β	*t*	*p*	*b*	β	*t*	*p*
Gender × age	0.00	0.08	–1.67	.09	0.01	0.15	–3.17	< .01	0.01	0.23	2.50	< .05	–0.00	–0.08	1.53	0.13
Gender × ethnicity																
Latino/Hispanic	0.06	0.01	0.66	0.51	0.08	0.02	0.84	0.40	–0.12	–0.02	–0.59	0.55	0.04	0.01	0.37	0.71
Middle Eastern	0.01	0.00	0.03	0.98	–0.27	–0.03	–1.29	0.20	0.42	0.03	1.04	0.30	0.16	0.02	0.78	0.43
Black/African	–0.22	–0.06	–2.15	< .05	–0.17	–0.05	–1.66	0.10	–0.28	–0.06	–1.21	0.22	0.49	0.13	4.23	<.001
Caribbean	–0.21	–0.01	0.47	0.64	–0.59	–0.03	–1.32	0.19	–0.40	–0.02	–0.47	0.64	–0.11	–0.01	–0.24	0.81
South Asian	0.18	0.02	0.81	0.42	0.24	0.02	1.06	0.29	–0.12	–0.01	–0.22	0.83	0.30	0.03	1.29	0.20
East Asian	–0.15	–0.02	–0.78	0.43	–0.08	–0.01	–0.41	0.68	0.15	0.02	0.51	0.61	0.02	0.00	0.11	0.92

*Note*. This table displays only interactions. Main effects for age and ethnicity across all well-being indicators can be found in Supplementary Table S3.

#### Gender and Ethnicity

When examining the interaction between gender and ethnicity, a significant two-way interaction emerged for relationship status satisfaction. Simple slopes analyses showed that compared to White men, Black men reported significantly higher relationship status satisfaction (*b* = 0.29, *t* = 3.64, *p* < .001, *R*^2^ = 0.003) while no significant relationship emerged when comparing White and Black women’s relationship status satisfaction (*b* = 0.07, *t* = 1.21, *p* = .22). Moreover, a two-way interaction emerged between gender and ethnicity for the desire for a partner such that Black women reported significantly higher desire for a partner relative to White women (*b* = 0.41, *t* = 5.99, *p* < .001, *R*^2^ = 0.01) while Black and White men showed no differences in their desire for a partner (*b* = –0.08, *t* = –0.85, *p* = .39). No significant interactions emerged between gender and ethnicity for life satisfaction or sexual satisfaction.

### Exploratory Analyses

We conducted exploratory analyses to preliminarily examine follow-up questions regarding explanations for gender differences in singles’ well-being. First, we examined relationship status as a moderator which is reported below. Second, we examined whether the gender and well-being link might be mediated by satisfaction in several life domains including friendship and family relationships. Given the concerns for replicability of the latter results, the interested reader can find them in the Supplementary Material.

To understand whether the gender differences in singles’ well-being are present across both partnered and single relationship status, we conducted interaction and simple main effects analyses examining the interaction between gender and relationship status (single or partnered) predicting our well-being indicators of relationship status satisfaction, life satisfaction, sexual satisfaction, but not partner desire as this measure was not administered to partnered participants. As Sample 2 and Sample 3 were the only samples that included partnered participants, we present the replicated results below (see Table 1 for sample sizes and participant demographics). We caution readers to consider these results as tentative given the considerably lower power relative to our within-group analyses.

Across both samples, a significant gender by relationship status interaction emerged for relationship status satisfaction (*b* = 0.30, *t* = 2.77, *p* < .01, *R*^2^ = 0.29; *b* = 0.04, *t* = 2.41, *p* < .05, *R*^2^ = 0.27). Simple slopes analyses revealed that for partnered individuals, there was no replicated significant association between gender and status satisfaction (Sample 3: *b* = 0.10, *t* = 1.33, *p* = .18; Sample 4: *b* = 0.02, *t* = 2.04, *p* = .04). Meanwhile, for singles, there was a significant association such that women reported greater status satisfaction than men (Sample 3: *b* = 0.40, *t* = 5.00, *p* < .001; Sample 4: *b* = 0.06, *t* = 5.31, *p* < .001). No significant gender by relationship status interactions emerged for life satisfaction or sexual satisfaction. Overall, our exploratory results suggest that gender differences in life and sexual satisfaction may not be unique to singlehood. However, gender differences in relationship status satisfaction appear unique to singlehood in that partnered men and women were roughly equally satisfied with their status, whereas single men were less satisfied with their status than single women.

## Discussion

Our study provides an overview of gender differences in singles’ well-being using a comprehensive set of well-being indicators. We found that, on average, women reported higher overall well-being including higher relationship status satisfaction, higher life satisfaction, higher sexual satisfaction as well as a lower desire for a partner. Overall, single women appear to be higher in their overall well-being compared to men. At a broader level, our findings appear to run counter to existing stereotypes regarding women as the uniquely unhappy gender in singlehood (Ward, 2020).

Our findings regarding life satisfaction are in line with work showing that women, generally, report higher life satisfaction at moderate to large effect sizes, as observed in countries around the world (Arrosa & Gandelman, 2016; Solé-Auró et al., 2018). Thus, although higher life satisfaction is not unique to single women per se, our research highlights that single women are not an exception to this phenomenon. Whereas life satisfaction is a domain general indicator of well-being, our other well-being indicators are more specifically tied to relationship status. Satisfaction with relationship status, sexual satisfaction, and desire for a romantic partner are closely tied to one’s relationship status, and women reported higher well-being across all these indicators. Moreover, our exploratory analyses suggested that the gender differences in status satisfaction may be unique to the singlehood context.

Why might single women report higher well-being? One potential reason is that women have stronger social support beyond romantic relationships. Indeed, the social support and life satisfaction link is well-documented (Diener et al., 2006), and evidence shows that women tend to have larger social networks than men (McLaughlin et al., 2010). This link is especially crucial for singles who report nonromantic social support as a key aspect of their well-being in singlehood (Sarkisian & Gerstel, 2016). Notably, single women in particular are more likely to report satisfaction with social support compared to single men (Stronge et al., 2019), which may be a particular resource for single women. For example, one study showed that singles who reported greater social life satisfaction also reported lower partner desire (Kislev, 2020), but this link was not significant for divorced men. Overall, women’s greater perceived social support could make women’s lives better both in singlehood and partnership.

However, there may be some relationship status related reasons why single women may be more content in terms of relationship status satisfaction. According to theoretical perspectives on heteronormativity, inequities within heterosexual relationships, including inequitable divisions of household labor and the deprioritization of women’s sexual pleasure, lead to more rewards for men and more costs for women in committed heterosexual partnerships (van Anders et al., 2022). Add to this that one traditional advantage for women in partnering with men—income—is dissipating as societies trend toward increasing pay equity (Kislev, 2019), and it appears possible that men’s lower relationship status satisfaction when single may be an accurate recognition that they have more to gain from partnering than do single women.

Our findings examining the intersection of gender, age, and ethnicity revealed that, for age interactions, older single men reported lower life satisfaction and lower sexual satisfaction relative to younger single men. In contrast, Hill Roy and colleagues (2023) found no significant differences between younger and older men but observed that older women (around age 48) with lower partner desire reported higher life satisfaction. It is unclear why there may be a difference in well-being between younger and older single men, but Carr and colleagues’ (2024) finding that lifelong single men in the United States reported the highest poverty rates relative to men in other marital statuses suggests that wealth could be one mechanism explaining older single mens’ lower well-being.

The finding that older single men report lower sexual satisfaction contradicts existing work showing that men above 60, collapsing across relationship status, report greater sexual satisfaction at moderate effect sizes (Træen et al., 2019), although our effect was small. While older men generally report greater sexual satisfaction than older women (Laumann et al., 2006; Træen et al., 2019), our results may be explained by gender differences in sexual activity, which is positively associated with sexual satisfaction (McNulty et al., 2016). Karraker and colleagues (2011) show that while married men in midlife (ages 44–59) to later life (ages 57–72) report greater sexual activity than married women, for widowed and never-married participants, women reported greater sexual activity. This suggests the lower sexual satisfaction among older single men in our data may reflect an aspect of our results that particularly pertains to sexual activity and gendered dynamics in singlehood.

Meanwhile, interactions with ethnicity showed that Black men reported higher relationship status satisfaction than White men. These differences may be explained by demographic expectations surrounding singlehood between Black and White adults. For example, due to the higher rate of divorce and separation among Black Americans relative to White Americans, marriage is considered to be less normative among Black Americans (Tucker & Taylor, 1989). As such, stigma surrounding singlehood may be less pronounced for Black Americans. However, why there are gender differences in these experiences is unclear as there is limited research examining the intersection of race, gender, and singles’ well-being (cf. Pudrovska et al., 2006) that can directly speak to our findings.

Moreover, single Black women reported a greater desire for a partner than single White women. These results may be explained by barriers in dating experienced by Black women. For example, Clarke (2011) examined Black women’s experiences in romance and found that they often reported guilt and frustration over their barriers to romance, creating a yearning for satisfying sexual lives. In addition, in Moorman’s (2020) interviews with single Black women, frustration with singlehood was one of the emerging themes in participants’ responses. These frustrations and barriers may create contexts whereby single Black women have a greater desire for a partner.

This study holds several strengths and limitations. The primary strength of our study is the large sample size and integration of multiple data sets that allowed for a well-powered assessment of gender differences in well-being. We also employed measures of well-being that have shown strong psychometric properties (Lehmann et al., 2015; Park & MacDonald, 2022). Importantly, we also examined singles’ experiences using a within-group approach, allowing us to highlight the nuances within singles’ well-being that could be overlooked when conducting between-group comparisons across singles and partnered people (Park et al., 2024).

However, there are important limitations of this study that could be addressed in future work. One major limitation of this study is the lack of nonbinary individuals. Although the option was provided in our surveys, only a small proportion of individuals identified as nonbinary, making their group statistically underpowered. Future work could employ stratified sampling to ensure that nonbinary participants are represented in the data. To fully understand gender dynamics in singlehood, moving beyond dichotomous distinctions of gender is necessary. Moreover, we only measured hedonic well-being, but not eudaimonic well-being or ill being, limiting our ability to fully examine singles’ well-being and holistically capture well-being as a construct. For example, Pudrovska and colleagues (2006) showed that women reported greater levels of single strain, or the degree of stress one feels as a single person, while in Girme et al.’s (2023) survey of singlehood research, they also note a need for work tapping into meaning and richness of life. Importantly, our data did not capture the marital histories of singles including whether singles were widowed, divorced, or never married. Evidence shows that important heterogeneity exists across these categories, such as in their well-being and personality (Park et al., 2022; Tessler et al., 2024), and as such, should be examined in future work. Furthermore, our study descriptively examined gender differences in singles’ well-being, but is limited in its ability to understand mechanisms of this relationship. Future studies could examine mechanisms in the gender and well-being link, like high-quality social support which could buffer the lower well-being for single men.

One important implication of our study is in understanding why most incels, or involuntary celibates, are men. Incels are a misogynistic extremist group who blame women for their failed romantic and sexual pursuits (Ging, 2019). Research is increasingly examining contributors to inceldom like loneliness and depression (Sparks et al., 2024). Our findings, showing that single men report lower well-being generally, may help explain men’s overrepresentation in inceldom. Our findings suggest that incel researchers could extend their work to include the experiences of single men more broadly as the story of unhappy single men appears to reach well beyond incels.

Overall, this study aimed to provide insight into gender differences in singles’ well-being. This work builds on existing research by providing a direct examination of gender differences across various well-being indicators. Our results show that gender differences in singles’ well-being do exist such that women appear to be higher in their overall well-being. Altogether, our study advances existing work to clarify the role of gender in singles’ well-being and provide future directions for understanding gender among the growing population of singles.

## Supplemental Material

sj-docx-1-spp-10.1177_19485506241287960 – Supplemental material for “Sisters Are Doin’ It for Themselves”: Gender Differences in Singles’ Well-Being Supplemental material, sj-docx-1-spp-10.1177_19485506241287960 for “Sisters Are Doin’ It for Themselves”: Gender Differences in Singles’ Well-Being by Elaine Hoan and Geoff MacDonald in Social Psychological and Personality Science
