# Supplementary material for: “Sisters Are Doin’ It for Themselves”: Gender Differences in Singles’ Well-Being
Source: Soc Psychol Personal Sci. 2024 Oct 24;16(6):610–9. doi: 10.1177/19485506241287960 (PMC12178563; doi:10.1177/19485506241287960)
Supplement: sj-docx-1-spp-10.1177_19485506241287960 – Supplemental material for “Sisters Are Doin’ It for Themselves”: Gender Differences in Singles’ Well-Being [file sj-docx-1-spp-10.1177_19485506241287960.docx]

**Supplementary Materials**

**“Sisters are Doin’ It for Themselves”: Gender Differences in Singles’ Well-being**

Table of Contents

**Table S1: Sample characteristics per sample**

**Table S2. Means, Standard Deviations, and Alpha Reliability Coefficients for each sample**

**Table S3: Linear Regression Analyses Across Well-being Indicators**

**Figure S1: Mean Well-being Outcomes Across Study Time Periods (2018 to 2023)**

**Supplementary Text 1: List of R Packages Used**

**Supplementary Text 2: Age and Ethnicity Main Effects**

**Supplementary Text 3: Exploratory Analyses: Life Domain Satisfaction**

| **Sample** | **Data Collection** | **Gender** | **Age** | **Sexual Orientation** | **Ethnicity** | **Study Description** |
| --- | --- | --- | --- | --- | --- | --- |
| **Sample 1** (n = 534) | April 2023 | 291 Men 231 Women 9 Non-binary | 29.26 (9.147) | 77% Heterosexual 5% Homosexual 12% Bisexual 1% Queer 1% Questioning 2% Pansexual | ─ | Study about sexual connections in singlehood |
| **Sample 2** (n = 380) | March 2023 | 140 Men 209 Women 9 Non-binary | 29.31 (9.96) | 71% Heterosexual 6% Homosexual 15% Bisesexual 1% Queer 2% Questioning 2% Pansexual 2% Asexual | ─ | Study about personality and well-being |
| **Sample 3** (n = 475) | January 2023 | 252 Men 217 Women 6 Non-binary | 28.73 (8.93) | 78% Heterosexual 5% Homosexual 8% Bisexual 2% Queer 3% Questioning 2% Pansexual 1% Asexual | 60% White 10% Latino/Hispanic 1% Middle Eastern 12% Black 4% Asian 3% Other | Study about personality and well-being |
| **Sample 4** (n = 747) | January 2021 | 376 Men 371 Women | 38.82 (11.66) | 86% Heterosexual 4% Homosexual 5% Bisexual 1% Queer 1% Questioning 1% Pansexual 1% Asexual | ─ | Study about singles' time use and well-being |
| **Sample 5** (n = 200) | November 2018 | 114 Men 84 Women 1 Non-binary | 33.73 (10.61) | 87% Heterosexual 3% Homosexual 7% Bisexual 1% Queer 1% Questioning 1% Pansexual 1% Asexual | 73% White 6% Latino/Hispanic 16% Black 4% Asian | Study about beliefs about relationships and singlehood |
| **Sample 6** (n = 489) | June 2021 | 243 Men 245 Women | 23.40 (6.24) | 77% Heterosexual 2% Homosexual 13% Bisexual 2% Queer 4% Questioning | 58% White 14% Latino/Hispanic 3% Middle Eastern 8% Black 2% South Asian 5% Other | Study about motivations for dating |

**Table S1. Sample characteristics per sample**

| **Sample 7** (n = 811) | November 2021 | 416 Men 390 Women | 24.64 (6.49) | 78% Heterosexual 4% Homosexual 10% Bisexual 1% Queer 3% Questioning 2% Asexual | 31% White 28% Latino/Hispanic 2% Middle Eastern 33% Black 4% Other | Study about motivations for dating |
| --- | --- | --- | --- | --- | --- | --- |
| **Sample 8** (n = 525) | January 2021 | 261 Men 264 Women | 24.67 (8.59) | 78% Heterosexual 3% Homosexual 12% Bisexual 1% Queer 5% Questioning | 71% White 5% Latino/Hispanic 7% Middle Eastern 2% Black 3% South Asian 7% Other | Study about singles' ideal partner characteristics |
| **Sample 9** (n = 864) | April 2020 | 428 Men 434 Women 2 Non-binary | 38.49 (11.76) | 81% Heterosexual 6% Homosexual 8% Bisexual 1% Queer 1% Questioning 1% Pansexual | 74% White 7% Latino/Hispanic 1% Middle Eastern 4% Black 3% South Asian 4% Other | Study about profiles of singles |
| **Sample 10** (n = 937) | December 2020 | 462 Men 473 Women 2 Non-binary | 37.76 (11.66) | 81% Heterosexual 6% Homosexual 7% Bisexual 1% Queer 1% Questioning 1% Pansexual 1% Asexual | 78% White 5% Latino/Hispanic 2% Middle Eastern 3% Black 1% Carribean 2% East Asian 3% Other | Study about singles' social goals |

*Note.* Total percentages and frequencies may not equate to the sample size as some individuals preferred to not report their gender, sexual orientation, and/or ethnicity.

| **Table S2. Means, Standard Deviations, and Alpha Reliability Coefficients for each sample** | | | | | | |  |  |  |  |
| --- | --- | --- | --- | --- | --- | --- | --- | --- | --- | --- |
|  | **Sample 1** | **Sample 2** | **Sample 3** | **Sample 4** | **Sample 5** | **Sample 6** | **Sample 7** | **Sample 8** | **Sample 9** | **Sample 10** |
|  | (n = 534) | (n = 380) | (n = 475) | (n = 747) | (n = 200) | (n = 489) | (n = 811) | (n = 525) | (n = 864) | (n = 937) |
| Satisfaction with Relationship Status | 1.48 (0.81) | 1.54 (0.87) | 1.60 (0.83) | 1.60 (0.88) | 1.65 (0.80) | 1.48 (0.80) | 1.56 (0.80) | 1.57 (0.76) | 1.53 (0.85) | 1.39 (0.84) |
|  | α = 0.91 | α = 0.92 | α = 0.91 | α = 0.93 | α = 0.88 | α = 0.90 | α = 0.89 | α = 0.90 | α = 0.93 | α = 0.92 |
| Life Satisfaction | 3.61 (1.43) | 3.32 (1.46) | 3.58 (1.47) | 3.54 (1.49) | 4.38 (1.51) | 3.61 (1.37) | 3.61 (1.48) | 3.73 (1.35) | 3.45 (1.43) | 3.33 (1.41) |
|  | α = 0.89 | α = 0.89 | α = 0.90 | α = 0.91 | α = 0.92 | α = 0.88 | α = 0.89 | α = 0.88 | α = 0.91 | α = 0.91 |
| Sexual Satisfaction | 3.04 (1.68) | 2.43 (1.66) | 2.82 (1.78) | 2.60 (1.66) | ─ | ─ | ─ | ─ | ─ | 2.47 (1.60) |
|  | α = 0.95 | α = 0.97 | α = 0.97 | α = 0.96 | ─ | ─ | ─ | ─ | ─ | α = 0.96 |
| Desire for a Partner | 4.57 (1.12) | ─ | ─ | 4.20 (1.71) | ─ | 4.37 (1.57) | 4.40 (1.60) | 4.38 (1.50) | 3.79 (1.73) | 4.13 (1.68) |
|  | α = 0.91 | ─ | ─ | α = 0.95 | ─ | α = 0.93 | α = 0.93 | α = 0.92 | α = 0.94 | α = 0.95 |

*Note.* Gender was coded as 1 = Man and 2 = Woman. The reference category for ethnicity was 1 = White/Caucasian.

**Figure S1. Mean Well-being Outcomes Across Study Time Periods (2018 to 2023)**

**Figure S1a.** Mean Relationship Status Satisfaction for all samples between 2018 and 2023.


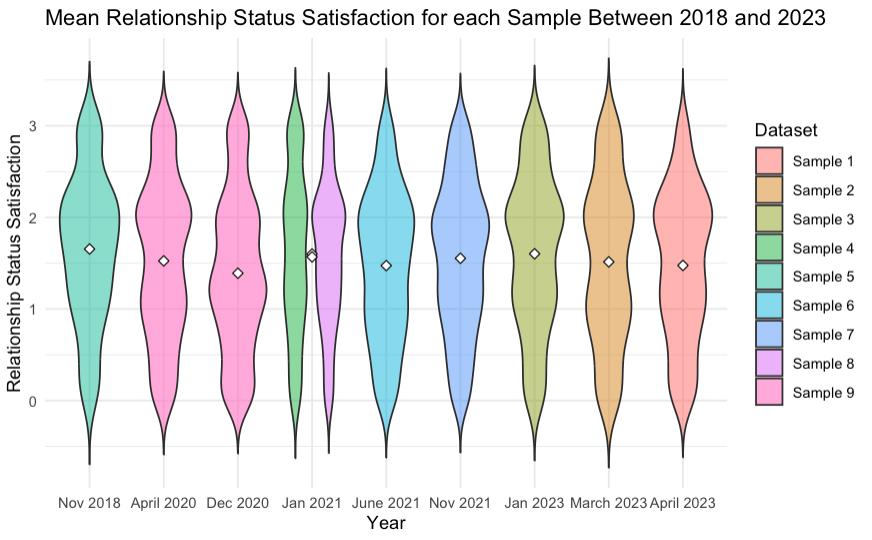


**Figure S1b.** Mean Life Satisfaction for all samples between 2018 and 2023.


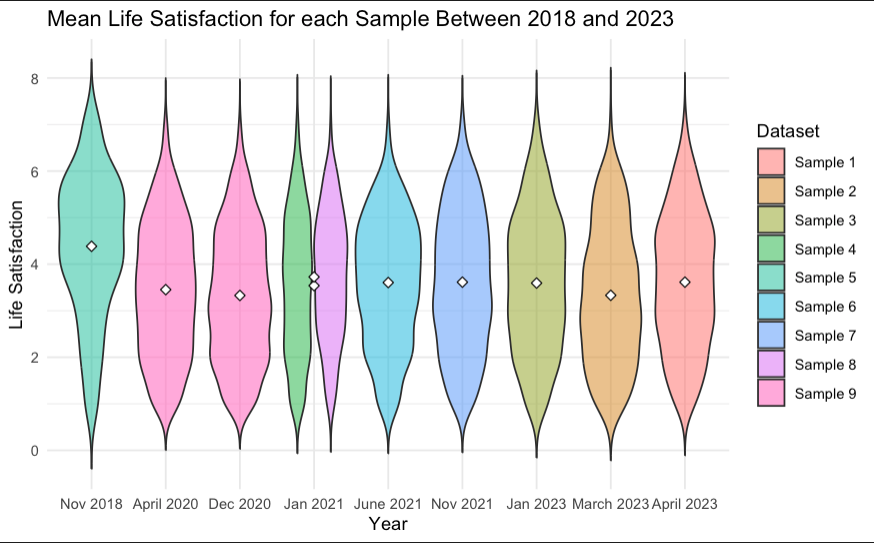


**Figure S1c.** Mean Sexual Satisfaction for all samples with available data between 2020 and 2023.


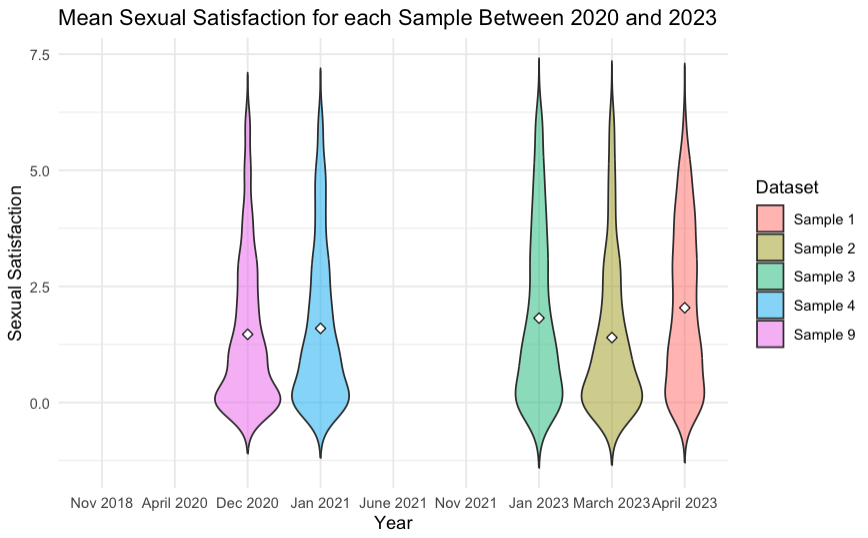


**Figure S1d.** Mean Desire for a Partner scores for all samples with available data between 2020 and 2023.


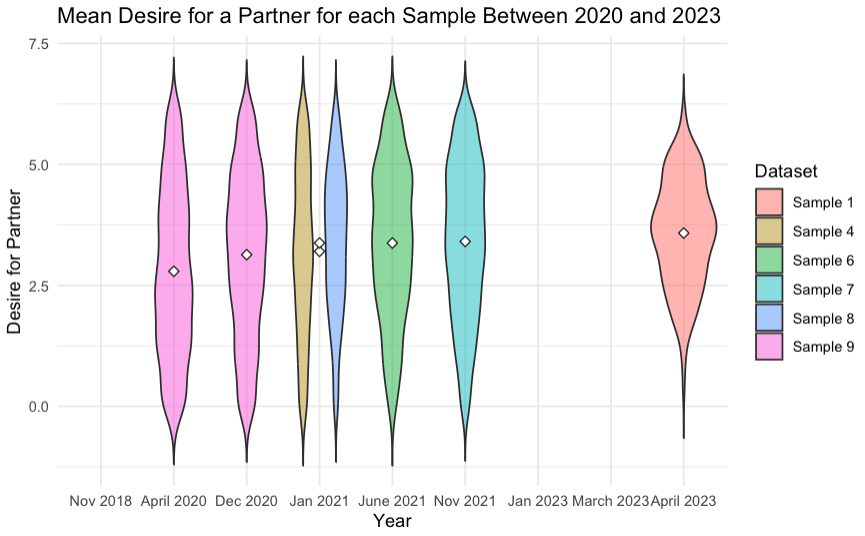


*Note*. Overall means of each sample are depicted through the white diamonds and distributions of values are visualized around each mean.

**Supplementary Text 1: List of R Packages Used**

Bates, D., Maechler, M., Bolker, B., & Walker, S. (2015). Fitting Linear Mixed-Effects Models

Using lme4. *Journal of Statistical Software*, *67*(1), 1-48.

<https://doi.org/10.18637/jss.v067.i01>.

Behrendt S (2023). *lm.beta: Add Standardized Regression Coefficients to Linear-Model-*

*Objects*. R package version 1.7-2, <https://CRAN.R-project.org/package=lm.beta>.

Chan, C., Chan, G. C., Leeper, T. J., and Becker, J. (2021). *rio: A Swiss-army knife for data file*

*I/O*. R package version 0.5.29.

Cinelli, C., Ferwerda, J., & Hazlett, C. (2021). *sensemakr: Sensitivity Analysis Tools for*

*Regression Models*. R package version 0.1.4,

<https://CRAN.Rproject.org/package=sensemakr>

Lenth, R (2023). *emmeans: Estimated Marginal Means, aka Least-Squares Means*. R package

version 1.8.7, https://CRAN.R-project.org/package=emmeans

Long, J. A. (2019). *Interactions: Comprehensive, User-Friendly Toolkit for Probing Interactions*.

R package version 1.1.0, <https://cran.r-project.org/package=interactions>.

Wickham H., Averick M., Bryan J., Chang W., McGowan L. D., François R., Grolemund

G., Hayes A., Henry L., Hester J., Kuhn M., Pedersen T. L., Miller E., Bache S. M.,

Müller K., Ooms J., Robinson D., Seidel D. P., Spinu V., … Yutani, H. (2019). Welcome

to the tidyverse. *Journal of Open Source Software*, *4*(43), 1686.

<https://doi.org/10.21105/joss.01686>

**Supplementary Text 2: Age and Ethnicity Main Effects**

**Age and Well-being Associations**

We conducted linear regressions with age as the primary predictor of well-being indicators to examine age and well-being associations amongst singles. As shown in Table S3, when relationship status satisfaction was entered as the outcome, results demonstrated that older singles reported significantly greater relationship status satisfaction (*b* = 0.03, *t* = 2.45, *p* < .05) while younger single participants reported significantly higher life satisfaction (*b* = –0.05, *t* = –3.53, *p* < .001). Moreover, older single participants also reported a lower desire for a partner (*b* = –0.14, *t* = –9.92, *p* < 0.001) as well as lower sexual satisfaction (*b* = –0.04, *t* = –2.30, *p* < .05).

**Ethnicity and Well-being Associations**

To examine well-being associations across ethnicities, we conducted regression analyses with ethnicity as our primary predictor and the well-being indicators as outcomes. These analyses showed that single participants identifying as Latino or Hispanic demonstrated high well-being overall such that they reported significantly higher relationship status satisfaction (*b* = 0.36, *t* = 7.52, *p* < .001) and life satisfaction (*b* = 0.32, *t* = 6.49, *p* < .001) compared to the reference group of White participants. Meanwhile, single participants identifying as Middle Eastern reported significantly lower relationship status satisfaction (*b* = –0.14, *t* = –1.45, *p* < .05) as well as a lower desire for a partner (*b* = 0.27, *t* = 2.71, *p* < .01). For Black/African and East Asian singles, a similar pattern of results emerged such that both groups of individuals reported significantly higher relationship status satisfaction (Black: *b* = 0.22, *t* = 4.45, *p* < .001; East Asian: *b* = 0.24, *t* = 2.50, *p* < .05) as well as significantly lower desire for a partner (Black: *b* = 0.19, *t* = 3.40, *p* < .001; East Asian: *b* = –0.22, *t* = –1.98, *p* < .05). For South Asian singles, no significantly associations emerged for the relationship-related well-being indicators, but South Asian singles reported significantly higher life satisfaction (*b* = 0.29, *t* = 2.56, *p* < .05). Meanwhile, no significant associations emerged for Caribbean individuals and any of the well-being indicators. It is plausible that the relatively low sample size of Caribbean singles (*n*  = 21) may result in a lack of statistical power.

**Supplementary Text 3: Exploratory Analyses: Life Domain Satisfaction**

To examine mechanisms underlying gender differences in well-being, we examined 1) whether there were gender differences in satisfaction across different life domains and 2) whether satisfaction in the domains that demonstrated gender differences would mediate the link between gender and life satisfaction. We conducted exploratory analyses using Sample 10 (n = 937) which included a measure of life domain satisfaction. This measure asked participants “How satisfied are you currently in the following aspects of your life?” rated on a 9-point scale from 1 (Not at all satisfied) to 9 (Extremely satisfied) and presented them with ten different life domains including independence (the freedom to make your own decisions), leisure, self-worth, friendships, family relationships, money, work/education, health, availability of romantic/sexual connections, religion. Importantly, we would like to note that the following findings should be taken with caution as they were post-hoc exploratory analyses that have yet to be replicated in a separate dataset. Moreover, there is reason to be cautious when interpreting cross-sectional mediations (Shrout, 2011).

**Gender Differences in Life Domain Satisfaction**

Firstly, we conducted independent samples t-tests to examine gender differences across satisfaction in these life domains. We found that single women reported significantly greater satisfaction with their independence (*t*(932) = -3.95, *p* < .001), friendships (*t*(932) = -3.01, *p* = .003), family relationships (*t*(932) = -2.12, *p* = .03), work/education (*t*(930) = -3.07, *p* = .002), and romantic/sexual relationships (*t*(913) = -3.80, *p* < .001) compared to single men. Meanwhile, single men reported significantly higher satisfaction with their leisure (*t*(927) = 2.58, *p* = .01) and health (*t*(926) = 2.83, *p* = .005). No significant gender differences were found for satisfaction with self-worth, (*t*(930) = -0.73, *p* = .47), money (*t*(927) = -0.41, *p* = .69), or religion (*t*(919) = -0.75, *p* = .45).

**Mediation by Life Domain Satisfaction**

Secondly, we conducted mediational analyses examining how satisfaction in the seven life domains that demonstrated significant gender differences, namely independence, friendships, family relationships, work/education, health, availability of romantic/sexual connections, leisure, and health, could mediate the relationship between gender and each of our four well-being indicators, including relationship status satisfaction, life satisfaction, sexual satisfaction, and desire for a partner. Amongst the seven life domains, only independence and the availability of romantic and sexual connections emerged as significant mediators across all four well-being indicators. Specifically, women reported greater satisfaction in their freedom to make their own decisions which contributed to their greater relationship status satisfaction (Indirect effect = 0.06, 95% CI = [0.03, 0.09]), life satisfaction (Indirect effect = 0.14, 95% CI = [0.07, 0.21]), sexual satisfaction (Indirect effect = 0.06, 95% CI = [0.03, 0.11]), and lower desire for a partner (Indirect effect = -0.03, 95% CI = [-0.07, -0.003]). Moreover, women reported greater satisfaction with the availability of romantic/sexual connections which contributed to their greater relationship status satisfaction (Indirect effect = 0.12, 95% CI = [0.06, 0.18]), life satisfaction (Indirect effect = 0.10, 95% CI = [0.05, 0.16]), sexual satisfaction (Indirect effect = 0.2890, 95% CI = [0.1423, 0.4439]), and lower desire for a partner (Indirect effect = -0.22, 95% CI = [-0.34, -0.11]).

**References**

Shrout, P. E. (2011). Commentary: Mediation Analysis, Causal Process, and Cross-Sectional

Data, *Multivariate Behavioral Research*, *46*(5), 852-860,

<https://doi.org/10.1080/00273171.2011.606718>
